# MicroRNA-1 acts as a tumor suppressor microRNA by inhibiting angiogenesis-related growth factors in human gastric cancer

**DOI:** 10.1007/s10120-017-0721-x

**Published:** 2017-05-10

**Authors:** Meng Xie, Dafydd Alwyn Dart, Ting Guo, Xiao-Fang Xing, Xiao-Jing Cheng, Hong Du, Wen G. Jiang, Xian-Zi Wen, Jia-Fu Ji

**Affiliations:** 1 Key Laboratory of Carcinogenesis and Translational Research (Ministry of Education/Beijing), Division of Gastrointestinal Cancer Translational Research Laboratory, Peking University Cancer Hospital & Institute, Beijing, China; 2 Cardiff China Medical Research Collaborative, Cardiff University School of Medicine, Cardiff, CF14 4XN UK

**Keywords:** miR-1, Gastric cancer, Vascular endothelial growth factor A, Angiogenesis

## Abstract

**Background:**

We recently reported that miR-1 was one of the most significantly downregulated microRNAs in gastric cancer (GC) patients from The Cancer Genome Atlas microRNA sequencing data. Here we aim to elucidate the role of miR-1 in gastric carcinogenesis.

**Methods:**

We measured miR-1 expression in human GC cell lines and 90 paired primary GC samples, and analyzed the association of its status with clinicopathological features. The effect of miR-1 on GC cells was evaluated by proliferation and migration assay. To identify the target genes of miR-1, bioinformatic analysis and protein array analysis were performed. Moreover, the regulation mechanism of miR-1 with regard to these predicted targets was investigated by quantitative PCR (qPCR), Western blot, ELISA, and endothelial cell tube formation. The putative binding site of miR-1 on target genes was assessed by a reporter assay.

**Results:**

Expression of miR-1 was obviously decreased in GC cell lines and primary tissues. Patients with low miR-1 expression had significantly shorter overall survival compared with those with high miR-1 expression (*P* = 0.0027). Overexpression of miR-1 in GC cells inhibited proliferation, migration, and tube formation of endothelial cells by suppressing expression of vascular endothelial growth factor A (VEGF-A) and endothelin 1 (EDN1). Conversely, inhibition of miR-1 with use of antago-miR-1 caused an increase in expression of VEGF-A and EDN1 in nonmalignant GC cells or low-malignancy GC cells.

**Conclusions:**

MiR-1 acts as a tumor suppressor by inhibiting angiogenesis-related growth factors in human gastric cancer. Downregulated miR-1 not only promotes cellular proliferation and migration of GC cells, but may activates proangiogenesis signaling and stimulates the proliferation and migration of endothelial cells, indicating the possibility of new strategies for GC therapy.

**Electronic supplementary material:**

The online version of this article (doi:10.1007/s10120-017-0721-x) contains supplementary material, which is available to authorized users.

## Introduction

Gastric cancer (GC) is the third commonest cause of cancer deaths worldwide [1]. In 2012, 42.54% of global new GC cases and 44.95% of global GC deaths occurred in China [1, 2]. Metastasis is the overwhelming cause of treatment failure in patients with GC. Therefore, a better understanding of the molecular mechanisms underlying distant metastasis would facilitate the development of novel effective therapeutic strategies for GC patients.

MicroRNAs (miRNAs) are endogenous, small noncoding RNAs that have an ability to promote or suppress the expression of many genes. They are involved in cell signaling pathways essential for tumor occurrence and progression, such as cell proliferation, mobility, apoptosis, and angiogenesis [3, 4].

Accumulating evidence has revealed aberrant expression of specific miRNAs in various malignant tumors, including GC. Our previous study analyzing miRNA sequencing data of GC from The Cancer Genome Atlas (TCGA) website revealed that miR-1 was markedly downregulated in GC compared with adjacent nonmalignant tissue samples. It ranked as the most decreased miRNA in the chromosome instability subgroup of GC, which accounted for half of the total tumors tested [5]. MiR-1, sharing a similar seed sequence with miR-206, was originally described as muscle specific, and has been shown to downregulate *MET*, *FOXP1*, *KRAS*, *PIK3CA*, and *NAIP*, which are important oncogenes relating to tumorigenic properties of various cancer types [6–10] and even tumor-associated macrophages [11]. Microarray analysis on biopsy samples from 90 GC patients and 34 healthy volunteers by Kim et al. [12] revealed that miR-1 was one of the mostly downregulated miRNAs in GC. Conversely, Liu et al. [13] found that serum miR-1 concentration was significantly high in GC patients compared with control individuals. Patients with high expression of serum miR-1 showed resistance to fluoropyrimidine-based chemotherapy [14]. Furthermore, no study has investigated the clinical significance of miR-1 expression in GC tissue samples.

Angiogenesis has a major function in tumor development and progression. In this setting, clinical data suggest that targeting angiogenesis by inhibiting angiogenic signaling pathways is an important therapeutic activity for many solid tumors, including GC. However, evidence of antitumor activity with antiangiogenic therapies leading to improved overall survival or progression-free survival in patients with metastatic GC is still limited [15, 16].

Vascular endothelial growth factor (VEGF)-A is a key regulator of angiogenesis [17]. In patients with GC, circulating VEGF-A levels are associated with increased tumor aggressiveness and reduced survival [18, 19]. The mechanisms that modulate the level of VEGF-A expressed by the producing cells are gradually being uncovered. Stahlhut et al. [20] demonstrated that miR-1 negatively regulated angiogenesis by suppressing *VEGFA* during zebrafish development. *VEGFA* and miR-1 are well conserved across all species, indicating that the same phenomenon may possibly occur during carcinogenesis.

Accordingly, in this study we characterized miR-1 expression in GC cell lines and primary GC tissues, and investigated its functional role in GC pathogenesis. We quantified miR-1 expression in both tumor and corresponding nontumor tissues, and analyzed the relationships between the levels of miR-1 expression and clinicopathological parameters in 90 Chinese patients with GC. We found that miR-1 was frequently downregulated in tumor tissues compared with corresponding nontumor tissues, and that low expression of miR-1 was correlated with poor prognosis. The effects of miR-1 on gastric carcinogenesis were evaluated by gain-of-function experiments. Overexpression of miR-1 significantly weakened malignant behavior of GC cells and tube formation of endothelial cells by directly suppressing *VEGFA* and *EDN1* expression.

## Materials and methods

### GC samples

Ninety matched GC and adjacent nontumor mucosal tissues (more than 5 cm laterally from the edge of the cancerous region) were collected from patients undergoing radical surgical resection at Peking University Beijing Cancer Hospital from January 2004 to December 2010. After gastrostomy, resected specimens were processed routinely for macroscopic pathological assessment, then harvested and frozen in −80 °C freezer. GC stage was classified according to the 2010 TNM classification recommended by the American Joint Committee on Cancer. Patient records were reviewed in the context of clinicopathology and follow-up information. All patients were tracked until March 2015. None of the patients received preoperative chemotherapy or radiation therapy before surgery. All samples were obtained with the patient’s informed consent. The Ethics Committee of Beijing Cancer Hospital approved this study.

### Cancer cell culture

One immortalized gastric mucosal epithelial cell line (GES-1) and 6 GC cell lines (SGC7901, MKN28, NCI-N87, BGC823, AGS, and HGC27) were cultured for miR-1 expression evaluation. The SGC7901 and BGC823 cell lines were acquired from the Cell Research Institute (Shanghai, China). The NCI-N87, AGS, and COS-7 cell lines were purchased from ATCC (Manassas, VA, USA), The HGC27 and MKN28 cell lines were obtained from the European Collection of Authenticated Cell Cultures (Porton Down, UK) and the Health Science Research Resources Bank (Tokyo, Japan) respectively. COS-7 cells were used for miR—mRNA interaction reporter assays only. Cell lines were cultured in Dulbecco’s modified Eagle’s medium (HyClone, Logan, UT, USA) supplemented with 10% (v/v) fetal bovine serum (Gibco-BRL, Invitrogen, Paisley, UK) and penicillin at 100 U/mL and streptomycin at 100 U/mL at 37 °C in a 5% CO_2_ incubator.

### 5-Aza-2′-deoxycyridine treatment

After being seeded at a density of 10^6^ cells per 10-cm dish on day 0, the GC cell lines were treated with freshly prepared 1 μM 5-aza-2′-deoxycyridine (5-aza-dC; Sigma, UK) for 24 h on days 1, 3, and 5. After each treatment, the medium was replaced with normal medium and harvested on day 6 for RNA extraction.

### Transient transfection

Logarithmically growing BGC823, SGC7901, AGS and HGC27 cells were seeded in a 10-cm dish (6 × 10^6^ cells per flask), and then transfected with 16 μg GV268-miR-1 (hsa-miR-1-1) or a nonspecific GV268-ctrl plasmid (GeneChem, China) for miR-1 overexpression and 30 nM antago-miR-1 or antago-miR negative control for miR-1 suppression (Ambion, Austin, TX, USA) with Lipofectamine 2000 reagent (Invitrogen, Carlsbad, CA, USA). The culture medium was replaced after 6 h, and then protein or RNA was extracted from subconfluent cells after transfection for 48 h.

### RNA extraction, reverse transcription, and quantitative real-time PCR

Total RNA was extracted from tissues or cultured cells with Trizol reagent. For messenger RNA (mRNA) expression analysis, RNA was reverse transcribed with Moloney murine leukemia virus reverse transcriptase (Invitrogen) with oligo(dT)_15_ primers. The complementary DNA was amplified with specific primers and Power SYBR Green PCR master mix (Applied Biosystems, Foster City, CA, USA). The glyceraldehyde 3-phosphate dehydrogenase (GAPDH) mRNA level was used as an internal normalization control. The sequences of the primers used were as follows: *VEGF-A* 5′-CCTGGTGGACATCTTCCAGGAGTACC-3′ (forward), 5′-GAAGCTCATCTCTCCTATGTGCTGGC-3′ (reverse); *EDN1* 5′-AGCCTCCTCTGCTCTTTCTGCTGGA-3′ (forward), 5′-CTTTTGTCTATGCCCCTGCAGCCTT-3′ (reverse); *MET* 5′-CTTTGTGAGCAGATGCGGAG-3′ (forward), 5′-GGTTTATCTTTCGGTGCCCAG-3′ (reverse); *GAPDH* 5′-ATGGGGAAGGTGAAGGTCG-3′ (forward), 5′-GGGGTCATTGATGGCAACAATA-3′ (reverse). TaqMan microRNA assays (Applied Biosystems) were used to quantify miR-1 (assay ID 002222) expression, and *SNORD48* (assay ID 001006) was used as the endogenous control. Gene-specific reverse transcription for miR-1 and *SNORD48* was carried out using about 500 ng of purified total RNA, 0.15 μL of 100 mM dNTPs (with dTTP), 1.5 μL MultiScribe reverse transcriptase (50 U/μL), 1.5 μL 10× reverse transcription buffer, 0.188 μL RNase inhibitor, 3.0 μL 5× TaqMan miRNA reverse transcription primer, and 4.162 μL nuclease-free water. A 15-µL reaction mixture was incubated for 30 min at 16 °C, 30 min at 42 °C, and 5 min at 85 °C to inactivate the reverse transcriptase. Then 1.33 μL reverse transcription product, 7.67 μL nuclease-free water, 10 μL TaqMan 2× universal PCR master mix (Applied Biosystems), and 1 μL TaqMan microRNA assay probe containing PCR primers and TaqMan probes were added and run in triplicate on an ABI Prism 7500 HT sequence detection system (Applied Biosystems) at 95 °C for 10 min followed by 40 cycles at 95 °C for 15 s and 60 °C for 1 min. Changes in miR-1 expression were normalized to *SNORD48* expression, and calculated with the 2^−ΔΔCt^ method. Each test was performed in triplicate.

### ELISA and Western blot assay

After 48 h of transfection, conditioned medium was removed and analyzed by ELISA for detection of secreted VEGF-A (human VEGF QuantiGlo ELISA kit, R&D Systems, Minneapolis, MN, USA). In parallel, whole-cell lysates were prepared with radioimmunoprecipitation assay lysis buffer supplemented with protein enzyme inhibitors. The protein concentration was determined by use of the bicinchoninic acid assay. Equal amounts of protein (20 ug per lane) were subjected to sodium dodecyl sulfate–polyacrylamide gel electrophoresis and transferred to poly(vinylidene difluoride) membranes. The membranes were blocked with 5% fat-free milk in tris(hydroxymethyl)aminomethane-buffered saline (TBS) (pH 7.5) for 1 h at room temperature, and then incubated with anti-VEGF-A (ab9570, Abcam), anti-entothelin 1 (ab113697, Abcam), anti-MET (sc-8307, Santa Cruz Biotechnology), or anti-GAPDH (sc-47724, Santa Cruz Biotechnology) antibodies overnight at 4 °C. After incubation with the appropriate horseradish peroxidase conjugated secondary antibody (Pierce) for 1 h at room temperature, membranes were incubated with enhanced chemiluminescence reagent and visualized in a Syngene gel documentation system.

### Cell proliferation and migration assay

To assay cancer cells for proliferation, they were seeded at a density of 3000 cells per well in a 96-well plate and maintained in regular medium. Cell growth was monitored by an IncuCyte^®^ live cell analysis system (Essen Instruments, Ann Arbor, MI, USA), where the cell proliferation was assessed by confluence measurements. Cell migration was assessed with a wound-healing assay using the IncuCyte system. For this, cancer cells were seeded at a density of 2 × 10^4^ cells per well in 96-well plates. A wound was made through confluent monolayer cells with a pin block, and cells were washed with 1× phosphate-buffered saline, and then cultured in regular medium. Photographs of cells were taken at 2-h intervals from two separate regions per well with a ×10 objective. Values from two regions of each well were pooled and averaged across all six replicates.

### Endothelial cell culture and tube formation assays

Human microvascular endothelial cells (HMVECs) were grown to confluence in 75-cm^2^ tissue culture flasks in medium 131 supplemented with microvascular growth supplement.

Supernatant from the BGC823 and AGS cells transfected with GV268-miR-1 or a nonspecific GV268-ctrl plasmid or the wild type was collected and centrifuged at 400g for 5 min. Purified supernatant then mixed with medium 131 in a ratio of 2:1, and which served as conditioned medium. HMVECs (3000 cells per well for proliferation and 10,000 cells per well for migration) were seeded in 96-well plates. After starvation, the supernatant was replaced with conditioned medium [21]. The migration of HMVECs was quantified by a wound healing assay with use of the IncuCyte system as described earlier.

For tube formation assay, 50 μL of liquefied Matrigel (BD Bioscience) was plated onto 96-well plates evenly and incubated for 30 min at 37 °C. A suspension of 5 × 10^4^ HMVECs was loaded on the top of the Matrigel. The conditioned medium was replaced after cell attachment. Following incubation (24 h at 37 °C, 5% CO_2_), pictures from six replicates of each group were captured with a Leica microscope, and the number of branches and nodes were quantitated by Wimasis Image Analysis. All the experiments were performed triplicate.

### Reporter assay

The human *VEGF-A* and *EDN1* 3′ untranslated region (UTR; 1938 and 1126 bp respectively) containing the putative binding sites of miR-1 (wild type) or an identical sequence with mutations of the miR-1 seed sequence (mutant) was amplified by PCR and then inserted into the firefly luciferase reporter vector pEZX-MT06. For luciferase assay, COS-7 cells were seeded in a 96-well plate at a density of 8000 cells per well. After overnight incubation, about 70% confluent cells were transiently transfected with 150 μL Opti-MEM (Gibco, USA) containing 0.15 μL Lipofectamine 3000 reagent (Invitrogen, USA) and 0.2 μL P3000 reagent, 100 ng luciferase reporter plasmids, and 120 ng GV268-miR-1 or GV268-ctrl plasmid per well. Cells were incubated for 6 h, and transfection medium was replaced with the 500 μL fresh regular medium. Luciferase activity was measured after incubation for 24 h with a dual-luciferase reporter assay system (GeneCopoeia, USA) by a microplate reader (Synergy HT, BioTek). Briefly, cells were lysed with 20 μL lysis buffer per well. The culture plate was incubated at −80 °C overnight, 100 μL firefly luciferase assay reagent was added to 20 μL lysate for the first measurement, and 100 μL *Renilla* luciferase reagent was added for the second measurement. The experiments were performed independently in duplicate.

### Statistical analysis

GraphPad Prism (version 6.01; GraphPad Software, La Jolla, CA, USA) was used to calculate the statistical significance of differences. Variance between three or more experiments and/or wells was calculated by analysis of variance and presented as the mean ± standard error of the mean. Statistical analyses of duplicate data (paired samples or unpaired samples) were determined by *t* tests or nonparametric tests. The overall survival was calculated with the Kaplan–Meier method and analyzed with the log-rank test. All statistical tests were two-sided, and *P* < 0.05 was considered statistically significant.

## Results

### Expression of miR-1 in human GC cell lines and primary GC samples

**Fig. 1:**
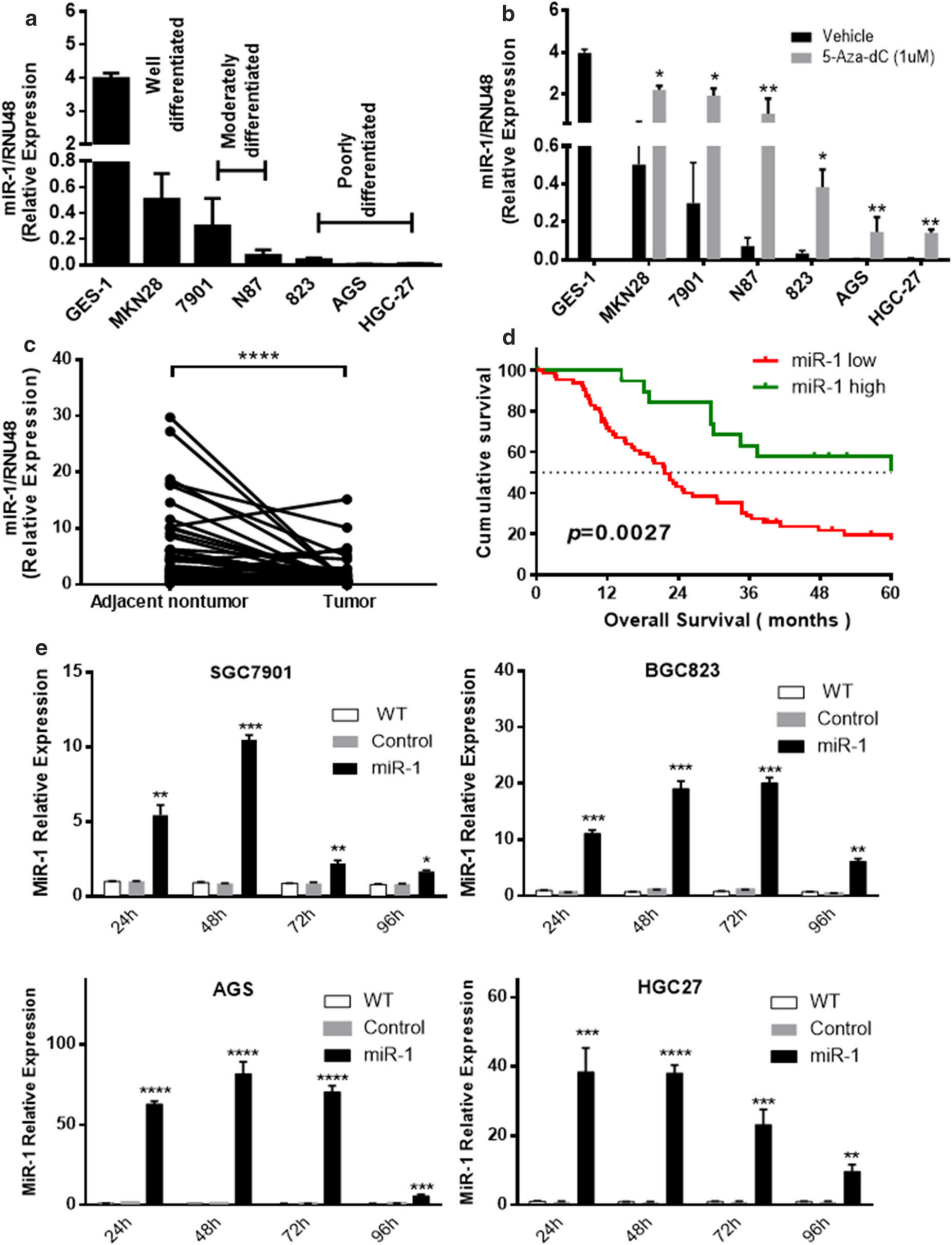
MiR-1 expression in cultured gastric cancer (GC) cells and primary GC tissues samples, and its correlation with prognosis of GC patients **a** MiR-1 expression in GC cell lines compared with the immortalized gastric cell line GES-1. **b** Quantitative PCR levels showing reexpression of mature miR-1 in GC cell lines after 5-aza-2′-deoxycyridine (*5-aza-dC*) treatment. **c** Relative expression levels of miR-1 in primary gastric tumors and adjacent nontumor tissues (*n* = 90). The data represent 2^−ΔΔCt^ expression values. The *P* value was calculated by a paired test. **d** Kaplan–Meier curves of overall survival for all patients with miR-1-high versus miR-1-low GC tissue. **e** Transfection efficiency of miR-1 in GC cells by qPCR . Mean ± standard deviation of three independent experiments. *WT* wild type, *P < 0.05, **P < 0.01, ***P < 0.001, ****P < 0.0001

Previously we reported that miR-1 was the most significantly downregulated miRNAs in GC on the basis of TCGA data [5]. To assess the miR-1 expression pattern in Chinese patients with GC, we firstly performed qPCR in GC cell lines and paired primary tissues from 90 GC patients with or without metastasis at diagnosis. As shown in Fig. 1a, miR-1 was obviously downregulated in GC-derived cell lines compared with normal stomach-derived cells (GES-1), and the expression level tended to be decreased with poor differentiation. After treatment with the demethylation agent 5-aza-dC, the level of miR-1 expression was restored significantly in all the GC cell lines examined compared with the wild-type cells, suggesting that DNA hypermethylation may account for miR-1 downregulation in GC cells (Fig. 1b). MiR-1 was also frequently downregulated in primary tumors compared with paired nontumor tissues (*P* < 0.0001) (Fig. 1c).

### Relationship between miR-1 expression and clinicopathological characteristics

We then analyzed the association of miR-1 expression status with clinicopathological features in GC patients. Recent reports tend to suggest that expression level changes between tumor and paired nontumor samples may be more correlated with cancer relapse and survival than expression levels in tumor samples alone, Huang et al. [22] reported that adjacent normal samples’ transcriptional levels likely provided complementary information on patient survival through systematic evaluation of transcription profiles of tumor adjacent to normal samples across multiple cancer cohorts using the TCGA pancancer data. Thus fold changes of tumor to nontumor miR-1 expression were adopted for evaluation—namely, a patient with a ratio less than one third was defined as having low expression, and a patient with a ratio greater than or equal to one third was defined as having high expression. As shown in Table 1, GC patients with low miR-1 expression showed a higher potential to develop vascular invasion, lymph node involvement, and distant metastasis (respectively 55.6% vs 30.8%, *P* = 0.033; 44.8% vs 30.4%, *P* = 0.040; and 81.8% vs 44.3%, *P* = 0.020). The proportion of patients with low miR-1 expression tended to increase with advanced TNM stages (*P* = 0.143). Kaplan–Meier survival curves showed that overall survival was worse in GC patients with low miR-1 expression compared with GC patients with high expression (*P* = 0.0027, Fig. 1d). Multivariate analysis was then performed on the following factors known to impact survival: tumor stage, tumor location, tumor size, vascular invasion, age, sex, and miR-1 expression. N category and M category demonstrated the most significant impact on survival (*P* = 0.004 and *P* < 0.0001). MiR-1 expression did not retain statistical significance with regard to survival (*P* = 0.108, Table 2). In contrast, when we considered the interaction between miR-1 expression and tumor stage, our multivariate analysis revealed the interaction between miR-1 expression and tumor stage was the only independent factor associated with worse prognosis of GC (*P* < 0.001).

**Table 1:** Relationship between miR-1 suppression and clinicopathological features in Chinese gastric cancer patients

	MiR-1 suppression		*χ* ^2^	*P* ^a^
	Positive	Negative		
Sex			0.600	0.549
Male	30 (46.9%)	34 (53.1%)		
Female	14 (53.8%)	12 (46.2%)		
Age (years)			0.319	0.750
≥60	23 (51.1%)	22 (48.9%)		
<60	21 (47.7%)	23 (52.3%)		
Tumor location			0.844	0.399
Cardia	11 (57.9%)	8 (42.1%)		
Noncardia	30 (46.9%)	34 (53.1%)		
Tumor size (cm)			1.845	0.065
≤4	1 (14.3%)	6 (85.3%)		
>4	40 (50.6%)	39 (49.4%)		
Histological differentiation			1.685	0.092^b^
Moderate	0 (0.0%)	3 (100.0%)		
Poor	43 (49.4%)	44 (50.6%)		
Vascular invasion			2.128	0.033
Absent	8 (30.8%)	18 (69.2%)		
Present	35 (55.6%)	28 (44.4%)		
Depth of invasion			0.6265	0.531^b^
T1	2 (66.7%)	1 (33.3%)		
T2–T4	42 (48.3%)	45 (51.7%)		
Lymph node involvement			1.655	0.040
No	7 (30.4%)	16 (69.6%)		
Yes	30 (44.8%)	37 (55.2%)		
Distant metastasis			2.294	0.022
M0	35 (44.9%)	43 (55.1%)		
M1	9 (81.8%)	2 (18.2%)		
TNM stage			6.114	0.057^c^
I	0 (0.0%)	1 (100.0%)		
II	2 (20.0%)	8 (80.0%)		
III	31 (47.0%)	35 (53.0%)		
IV	9 (81.8%)	2 (18.2%)		

^a^ *χ* ^2^ test

^b^Fisher–Kruskal–Wallis test

^c^Linear correaltion coefficient test

**Table 2:** Results of univariate and multivariate Cox proportional hazards regression analysis for overall survival of Chinese gastric cancer patients

Parameter	Univariate		Multivariate	
	5-year survival rate (%) (mean ± SE)	*P*	Relative risk^a^	*P*
Depth of invasion		0.284	0.707 (0.088–5.673)	0.696
T1	60.00 ± 0.00			
T2–T4	31.44 ± 2.25			
Lymph node involvement			3.215 (1.201–8.601)	0.004
No	48.74 ± 3.68	0.000		
Yes	26.65 ± 2.35			
Distant metastasis		0.002	3.592 (1.335–9.667)	0.000
M0	31.81 ± 2.60			
M1	15.86 ± 2.62			
MiR-1 expression		0.003	1.961 (0.458–2.019)	0.108
Low	36.63 ± 2.05			
High	27.69 ± 2.14			
Tumor size (cm)		0.119	1.525 (0.329–7.065)	0.378
≤4	44.45 ± 8.99			
>4	31.11 ± 2.29			
Vascular invasion		0.042	1.178 (0.527–2.634)	0.149
Absent	42.03 ± 4.14			
Present	29.21 ± 3.06			
Tumor location		0.003	1.408 (0.762–2.604)	0.395
Low	36.63 ± 2.05			
High	27.69 ± 2.14			
Sex		0.213	1.222 (0.589–2.536)	0.590
Male	34.29 ± 2.61			
Female	26.94 ± 4.20			
Age (years)		0.040	2.042 (0.965–2.425)	0.082
≤60	34.95 ± 3.24			
>60	28.44 ± 3.00			

*SE* standard error

^a^The 95% confidence interval is given in *parentheses*

### Overexpression of miR-1 suppressed proliferation and migration of GC cells

**Fig. 2:**
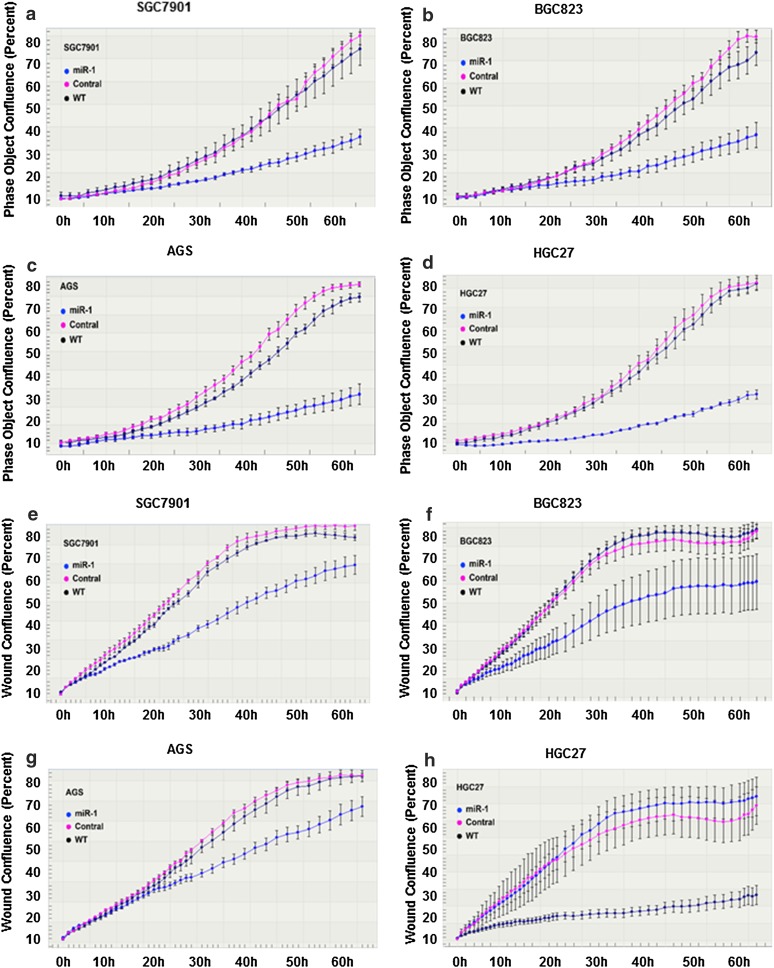
Overexpression of miR-1 suppressed the proliferation and migration capability of GC cells. **a**–**d** Cell growth curves. The *y*-axis is a label-free measure of cell confluence used for the IncuCyte ZOOM live cell imaging system to assess the cell growth. **e**–**h** Wound-healing curves. Cell motility was monitored by the IncuCyte ZOOM live cell imaging system. *WT* wild type

SGC7901, BGC823, AGS, and HGC27 cells were transfected with GV268-miR-1 plasmid, and the change in miR-1 expression at different time points was assessed by qPCR (Fig. 1e). Following transfection, cell proliferation and wound healing assays were performed with the IncuCyte system. The results showed that miR-1 significantly inhibited both cell proliferation (Fig. 2a–d, *P* < 0.05) and cell mobility (Fig. 2e–h) in all the GC cells examined. These results further indicate that miR-1 might act as a tumor suppressor in GC.

### MiR-1 inhibited VEGF-A, EDN1, and MET expression in GC cells

**Fig. 3:**
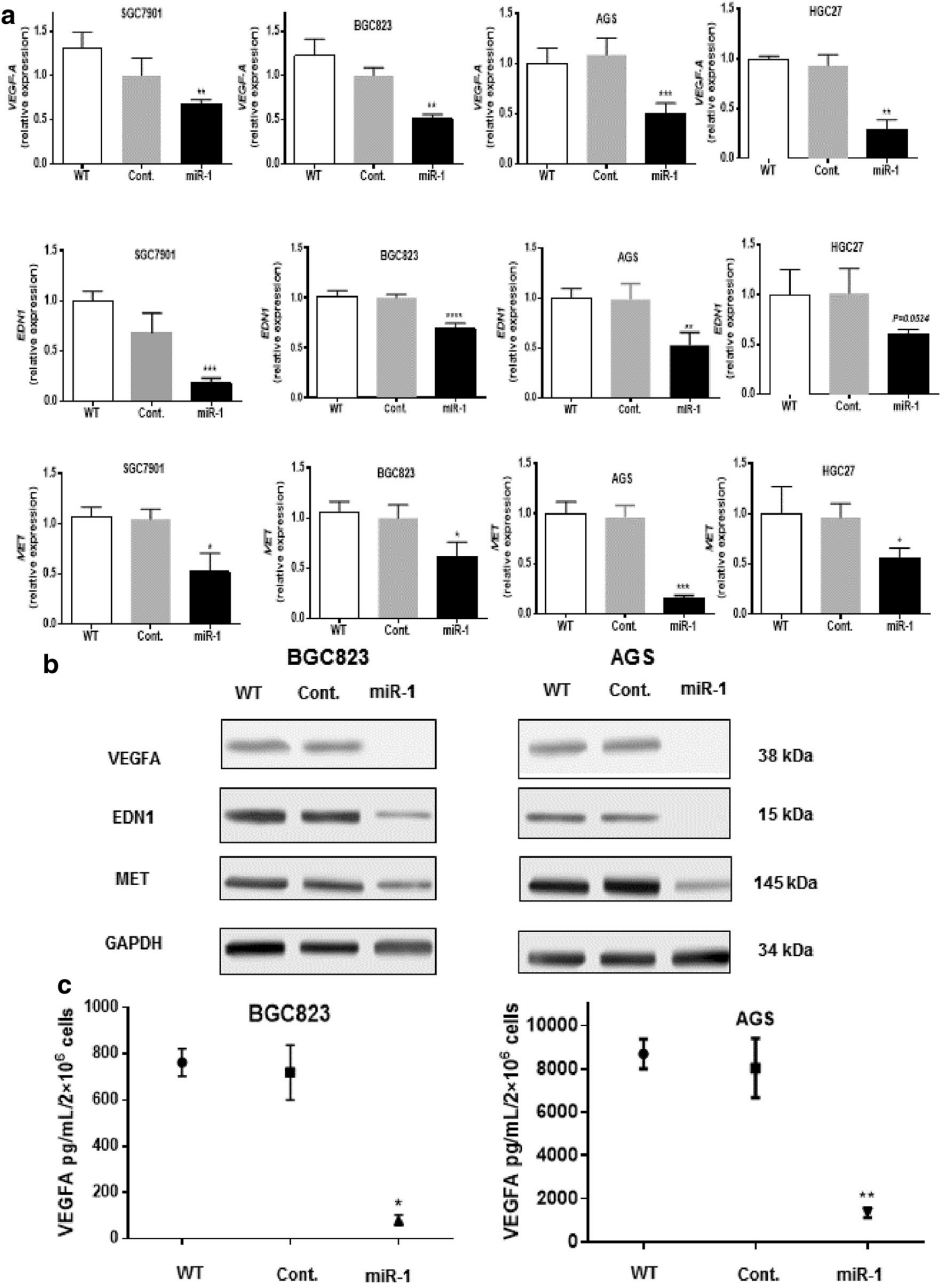
MiR-1 inhibited angiogenesis-related factors at both the messenger RNA level and the protein level. **a** Quantitative reverse transcription PCR assay. *P* values were determined by an unpaired two-sided *t* test. **b** Western blotting. **c** Relative protein levels of vascular endothelial growth factor A (*VEGF-A*) in supernatant of gastric cancer cells transitorily transfected with pri-miR-1 plasmid determined by ELISA. *Cont.* control, *WT* wild type, *P < 0.05, **P < 0.01, ***P < 0.001, ****P < 0.0001

MiRNA plays its role through regulating target gene expression by translational repression or degradation of mRNA in a sequence-specific manner. The TargetScanHuman (http://www.targetscan.org) algorithm predicted that *VEGF-A*, *EDN1*, and *MET* were directly targeted by miR-1. Furthermore, a protein array assay showed that these three targets were downregulated in miR-1-overexpressed cells compared with control cells (data not shown). Thus, we chose *VEGF-A*, *EDN1*, and *MET* for our further analysis. To investigate the direct effect of miR-1 on these predicted target genes in GC cells, the expression change of these genes was analyzed by qPCR and Western blotting at 48 h after miR-1 transfection. As shown in Fig. 3a and b, ectopic expression of miR-1 significantly inhibited VEGF-A, EDN1, and MET expression in GC cell lines at both the transcription level and the protein level. Considering VEGF-A is a paracrine growth factor, and the protein level in culture medium derived from BGC823 and AGS cells transfected with miR-1 or control was also assessed by ELISA. As we expected, the result was similar to that from Western blotting (Fig. 3c). We used antago-miR-1 to knock down its expression in highly differentiated MKN28 tumor cells and immortalized GES-1 gastric epithelial cells, both of which naturally express a relatively high level of miR-1. We found that transfection of MKN28 and GES-1 cells with antago-miR-1 caused a more than tenfold decrease in miR-1 expression compared with the negative control or the wild type (see Online Resource 3a in the electronic supplementary material). We further tested the changing level of VEGF-A, EDN1, and MET after miR-1 knockdown. The results revealed that inhibition of miR-1 expression significantly enhanced the expression of VEGF-A, EDN1, and MET at both the mRNA level and the protein level (see Online Resource 3b and c in the electronic supplementary material).

### MiR-1 interacts with a putative binding site in the *VEGF-A* and *EDN1* 3′ UTR

**Fig. 4:**
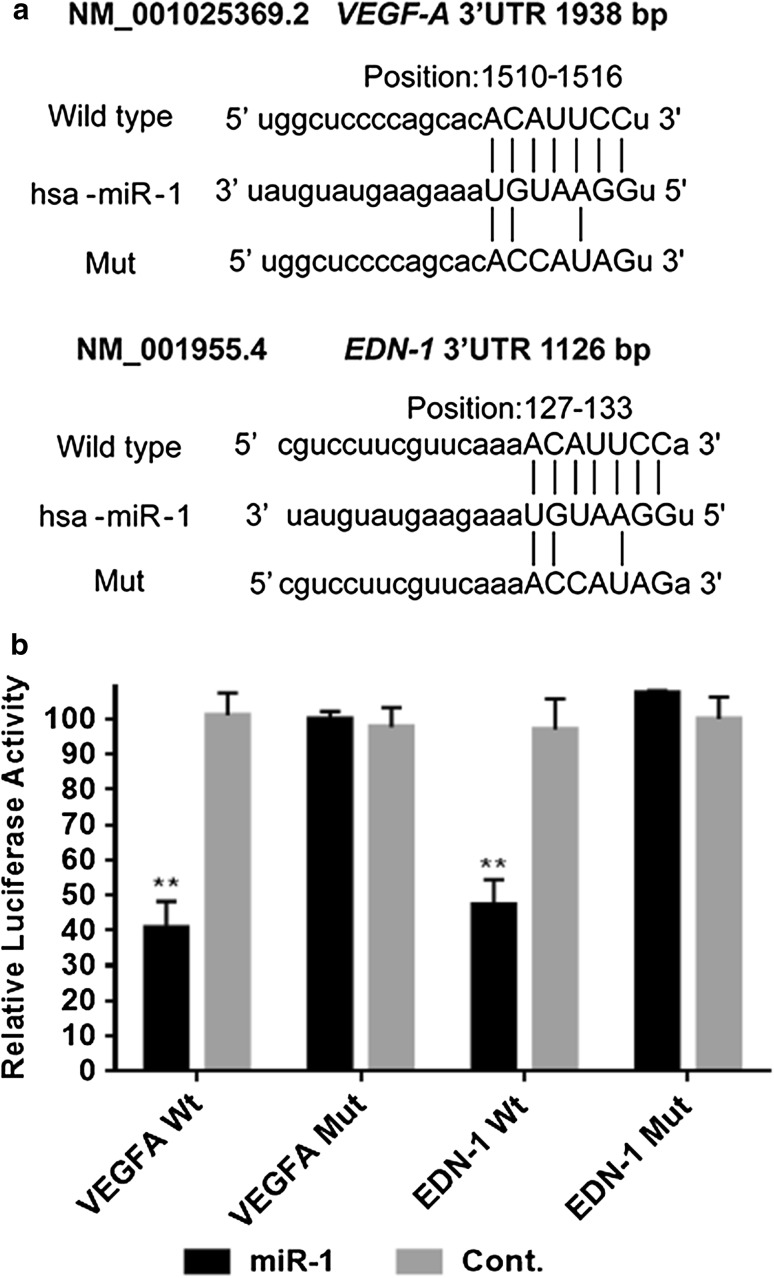
MiR-1 inhibits the expression of *VEGF-A* and *EDN1* 3′ untranlated region (*UTR*)-integrated luciferase reporter genes. **a** The target sites for miR-1 in the *VEGF-A* (NM_001025369.2), and *EDN1* (NM_001955.4) 3′ UTR were identified with the TargetScan database. **b** Luciferase reporter assay using the vector encoding the full-length 3′ UTR of *VEGF-A*, and *EDN1* 3′ UTR messenger RNA. The *Renilla* luciferase values were used to normalize firefly luciferase values. *Cont.* control, * Mut* mutant, *Wt* wild type, *P < 0.05, **P < 0.01

To determine whether miR-1 can inhibit *VEGF-A* and *EDN1* expression by targeting their binding sites in their 3′ UTRs, the PCR product containing each intact target site or mutant site of the miR-1 seed recognition sequence (Fig. 4a) was inserted into the luciferase reporter vector. COS-7 cells were transfected with these plasmids together with the GV268-miR-1 plasmid or GV268-ctrl plasmid. *VEGF-A* and *EDN1* firefly luciferase activity normalized to *Renilla* luciferase activity was significantly reduced in cells co-transfected with miR-1 (*P* < 0.05), but such reduction was not found on negative control transfection (Fig. 4b). On the other hand, the reporter vector lacking the miR-1 recognition site (mutant) fully rescued the miR-1 repression of both *VEGF-A* and *EDN1* luciferase activity (Fig. 4b), indicating that miR-1 directly and specifically interacts with the target site in the *VEGF-A* and *EDN1* 3′ UTRs.

### MiR-1 suppressed HMVEC proliferation, migration, and tube formation

**Fig. 5:**
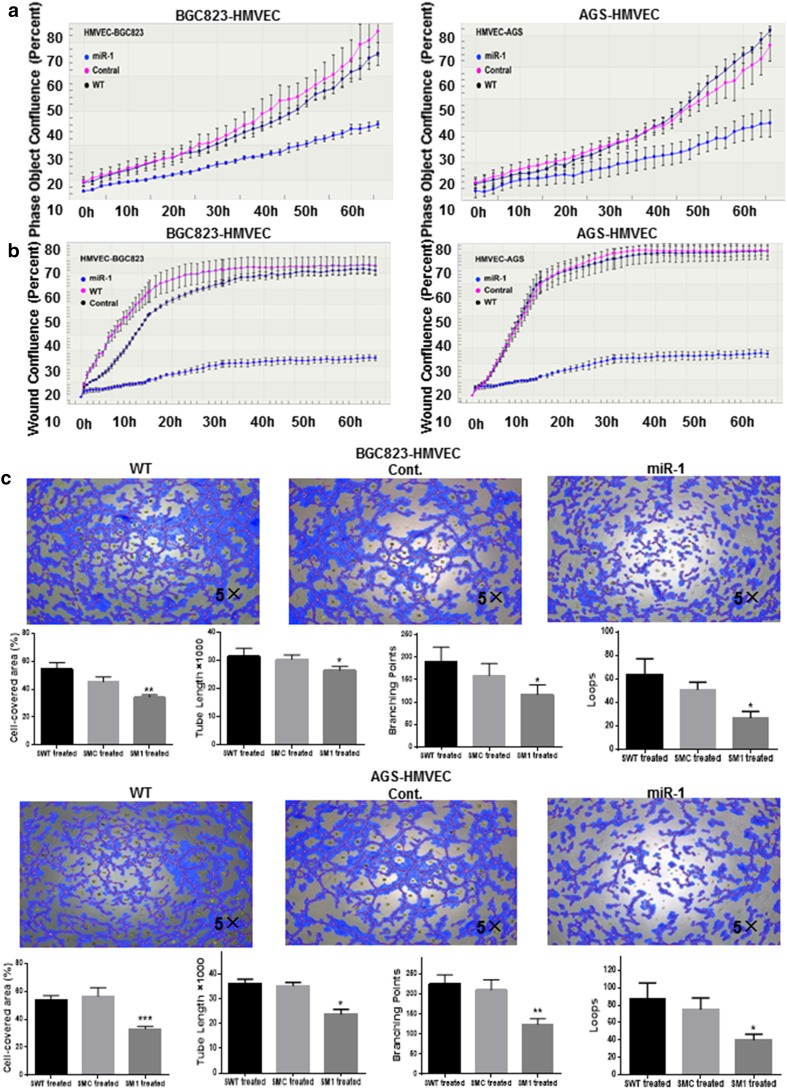
Effects of miR-1 on the proliferation, migration and tube formation of human microvascular endothelial cells. After both BGC823 and AGS cells had been transfected with GV268-miR-1 or GV268-ctrl plasmid respectively, the conditioned medium was collected and the effects of conditioned medium on human microvascular endothelial cell proliferation (**a**), migration (**b**), and tube formation (**c**) were assessed. The cell-covered area, tube length, branching points, and loops of tube formation were determined by WimTube from Wimasis Image Analysis (*n* = 3). One-way analysis of variance and Dunnett’s test were applied for analysis. *Cont.* control, *HMVEC* human microvascular endothelial cell, *WT* wild type, *P < 0.05, **P < 0.01, ***P < 0.001

Since VEGF-A and EDN1 have been implicated in the angiogenesis and metastasis [23, 24], and the expression of both VEGF-A and EDN1 was inhibited by miR-1 in our study, it is possible that miR-1 might participate in angiogenesis. To prove this assumption, we next investigated the effect of conditioned medium collected from BGC823 and AGS cells transfected with GV268-miR-1 or GV268-ctrl plasmid on endothelial cell proliferation, wound healing, and tube formation. As expected, HMVECs showed remarkable inhibition of cell growth, migration, and tube formation when cultured in conditioned medium derived from the miR-1-transfected cells compared with control ones (*P* < 0.05, Fig. 5).

## Discussion

Previous findings obtained from miRNome analysis of GC patients from TCGA revealed that miR-1 was the most downregulated miRNA in GC (see Online Resource 1 in the electronic supplementary material). In this study, qPCR analysis revealed that loss of miR-1 expression was frequently observed in primary gastric tumors compared with adjacent normal tissues, which was consistent with the data observed in breast, lung, colon, and hepatocellular carcinoma [7–10]. Intriguingly, some studies suggested an upregulation of plasma miR-1 in GC patients, including those who developed resistance to chemoagents [13, 14], by comparison with healthy individuals. However, there are increasing reports that miRNAs may be exported via an extracellular-vesicle-based active efflux mechanism, resulting in circulating miRNAs [25, 26].

In this study, we also observed an association between miR-1 expression and clinicopathological factors. Low miR-1 expression was positively related to lymph node involvement, vascular invasion, and distant metastasis. Furthermore, downregulation of miR-1 was negatively associated with 5-year survival rate. These results were consistent with the negative prognostic value of downregulated miR-1 expression in prostate cancer [27], colon cancer [28], and breast cancer [29]. In our multivariate analysis, low miR-1 expression alone exerted a marginal effect on the prognosis of GCs. Intriguingly, the interaction between miR-1 and stage was an independent factor for GC prognosis in the multivariate model, indicating that a strong association between miR-1 expression and tumor stage plays a profound role in GC pathogenesis. Hence, further investigations are warranted in a larger cohort.

Previously, methylation-mediated silencing of miR-1 was found in hepatocellular carcinoma [7], prostate cancer [27], and colorectal cancer [30]. We treated GC cell lines with a demethylating agent, 5-aza-dC, and assessed miR-1 expression by qPCR. Our data revealed a remarkable upregulation of mature miR-1 expression in all the GC cell lines examined, which is in accordance with previous findings, indicating hypermethylation may partly contribute to miR-1 suppression in GC.

Since miR-1 was downregulated in most of the GC cell lines, our gain-of-function experiments demonstrated that overexpression of miR-1 in GC cells attenuated the evil character of GC cells, such as proliferation and invasiveness, significantly compared with control groups. To identify the potential target of miR-1, we combined a bioinformatics prediction (TargetScanHuman) and a protein array assay. We selected three candidates: *VEGF-A*, *EDN1*, and *MET*. Western blot, qRT-PCR, and ELISA confirmed that overexpression of miR-1 in four GC cell lines significantly decreased VEGF-A, EDN1, and MET expression. We also knocked down miR-1 in MKN28 and GES-1 cells using antago-miR-1, and showed that inhibition of miR-1 expression significantly enhanced expression of MET and VEGF-A at the mRNA level and the protein level. These results were supported by the negative correlation between miR-1 expression and mRNA expression of *MET* and *VEGF-A* in our Chinese cohort (see online resource 2a and b in the electronic supplementary material) as well as the GC patients recruited in TCGA (see Online Resource 2d and e in the electronic supplementary material). *EDN1* exerted a weak correlation with miR-1 expression (see Online Resource 2c and f in the electronic supplementary material), however, the correlation failed to reach statistical significance in both cohorts. It may be explained by a more complicated molecular regulation in tissue samples than in vitro cell experiments. Taken together, our data imply a possible major role of miR-1 in downregulating *VEGF-A* and *MET* rather than *EDN1*. MET overexpression enhanced anchorage-independent growth, tumorigenesis, and experimental metastasis in vivo [31]. Furthermore, MET kinase inhibitors abolished cancer growth and reduced the tumor-associated angiogenesis in experimental tumor [32]. We found miR-1 directly targeted *MET*, which was supported by a recent report [33]. In addition, we identified *VEGF-A* and *EDN1* as two direct targets of miR-1. These findings could partially explain why overexpression of miR-1 could suppress the aggressiveness of GC.

VEGF-A, EDN1, and MET [34–36] are all major angiogenic factors involved in development and maintenance of blood vessels. We thus hypothesized that GC cells may downregulate miR-1 expression to tilt the balance toward stimulatory angiogenic factors to drive vascular growth, resulting in the development of GC metastasis. In this study, overexpression miR-1 in GC cells affected the endothelial cell tubular formation activity in a co-culture system study, implying that miR-1 may modulate gastric-tumor-related angiogenesis by regulating VEGF-A and EDN1. Recent studies have shown that EDN1 [37] and VEGF-A [38] promoted tumor progression via an angiogenesis-independent action of epithelial–mesenchymal transition, which may provide a plausible explanation for our observations that miR-1 inhibited the proliferation and migration of GC. Hence, our results suggest that loss of miR-1 expression was significantly correlated with metastasis and poor prognosis in primary GC. Further study will be warranted to confirm the antiangiogenetic biological effect of miR-1 and to determine whether miR-1 is directly involved in epithelial–mesenchymal transition in GC by use of an animal model.

Collectively, low miR-1 expression is strongly associated with a poorer prognosis in patients with GC as well as metastasis progression. These phenomena may be explained by our observation that aberrant expression of miR-1 direct targets, *VEGF-A*, *EDN1*, and *MET*, enhanced GC progression and stimulated angiogenesis. Moreover, targeting angiogenesis therapy is an effective component of the treatment strategy for cancer patients, but its efficiency is challenged by rapidly rising tumor resistance and limited improvements in overall survival. Thus, we propose miR-1 as an additional target to improve antiangiogenesis therapy in GC.

## Electronic supplementary material

Below is the link to the electronic supplementary material.

Supplementary material 1 (PDF 149 kb) Online Resource 1. MiR-1 was the most markedly downregulated microRNA (miRNA) in gastric cancer (*GC*) according to the heatmap illustrating miRNome profiles from 295 gastric cancer cases from The Cancer Genome Atlas (*TCGA*). The color code represents log_10_ of the ratio of miRNA expression means of the tumor sample group and the normal tissue group. The brighter the green color, the greater the decrease in miRNA expression in the tumor sample group compared with the normal tissue group. The *columns* represent four different molecular subtypes of GC: Epstein–Barr virus infected (*EBV*), chromosomal instability (*CIN*), genomically stable (*GS*), and microsatellite instability (*MSI*). The *rows* denote different miRNAs

Supplementary material 2 (PDF 188 kb) Online Resource 2. MiR-1 expression was negatively correlated with **a** *VEGF-A* [*R* = −0.23, 95% confidence interval (CI) −0.42 to −0.03], **b** *MET* (*R* = −0.21, 95% CI −0.40 to −0.01), and **c** *EDN1* (*R* = −0.18, 95% CI −0.38 to 0.03) expression in Chinese patients with gastric cancer. MiR-1 expression also exhibited negative correlation with **d** *VEGF-A* (*R* = −0.35, 95% CI −0.46 to −0.24), **e** *MET* (*R* = −0.18, 95% CI −0.30 to −0.06), and **f** *EDN1 *(*R* = −0.06, 95% CI −0.18 to −0.0)) in the GC patients from The Cancer Genome Atlas cohort

Supplementary material 3 (PDF 69 kb) Online Resource 3. Inhibition of miR-1 increases expression of angiogenesis-related factors at both the messenger RNA level and the protein level. **a** Transfection with antago-miR-1 inhibited miR-1 expression in MKN28 and GES-1 cells. Mean ± standard deviation of three independent experiments. **b** Quantitative PCR assay. *P* values were determined by an unpaired two-sided *t* test. **c** Western blotting. *Cont.* control, *CTRL*, control, *WT*, wild type, *P < 0.05, **P < 0.01, ***P < 0.001, ****P < 0.0001

## References

[1] Torre LA, Bray F, Siegel RL, Ferlay J, Lortet-Tieulent J, Jemal A (2015). Global cancer statistics, 2012. CA Cancer J Clin.

[2] Ferlay J, Soerjomataram I, Ervik M, Dikshit R, Eser S, Mathers C, Rebelo M, Parkin DM, Forman D, Bray, F. GLOBOCAN 2012 v1.0, cancer incidence and mortality worldwide: IARC CancerBase no. 11. Lyon: International Agency for Research on Cancer; 2013. Available from http://globocan.iarc.fr 10.1002/ijc.29210 25220842

[3] Lim LP, Lau NC, Garrett-Engele P, Grimson A, Schelter JM, Castle J (2005). Microarray analysis shows that some microRNAs downregulate large numbers of target mRNAs. Nature.

[4] Vasudevan S, Tong Y, Steitz JA (2007). Switching from repression to activation: microRNAs can up-regulate translation. Science.

[5] Xie M, Dart DA, Owen S, Wen X, Ji J, Jiang W (2016). Insights into roles of the miR-1, -133 and -206 family in gastric cancer (review). Oncol Rep.

[6] Yan D, Dong Xda E, Chen X, Wang L, Lu C, Wang J (2009). MicroRNA-1/206 targets c-Met and inhibits rhabdomyosarcoma development. J Biol Chem.

[7] Datta J, Kutay H, Nasser MW, Nuovo GJ, Wang B, Majumder S (2008). Methylation mediated silencing of microRNA-1 gene and its role in hepatocellular carcinogenesis. Cancer Res.

[8] Liu R, Li J, Lai Y, Liao Y, Liu R, Qiu W (2015). Hsa-miR-1 suppresses breast cancer development by down-regulating K-ras and long non-coding RNA MALAT1. Int J Biol Macromol.

[9] Zhao Q, Zhang B, Shao Y, Chen L, Wang X, Zhang Z (2014). Correlation between the expression levels of miR-1 and PIK3CA in non-small-cell lung cancer and their relationship with clinical characteristics and prognosis. Future Oncol.

[10] Xu X, Wu X, Jiang Q, Sun Y, Liu H, Chen R (2015). Downregulation of microRNA-1 and microRNA-145 contributes synergistically to the development of colon cancer. Int J Mol Med.

[11] Liu C, Wang J, Zhang X (2014). The involvement of MiR-1-clathrin pathway in the regulation of phagocytosis. PLoS One.

[12] Kim CH, Kim HK, Rettig RL, Kim J, Lee ET, Aprelikova O (2011). miRNA signature associated with outcome of gastric cancer patients following chemotherapy. BMC Med Genomics.

[13] Liu R, Zhang C, Hu Z, Li G, Wang C, Yang C (2011). A five-microRNA signature identified from genome-wide serum microRNA expression profiling serves as a fingerprint for gastric cancer diagnosis. Eur J Cancer.

[14] Huang D, Wang H, Liu R, Li H, Ge S, Bai M (2014). miRNA27a is a biomarker for predicting chemosensitivity and prognosis in metastatic or recurrent gastric cancer. J Cell Biochem.

[15] Aprile G, Ongaro E, Del Re M, Lutrino SE, Bonotto M, Ferrari L (2015). Angiogenic inhibitors in gastric cancers and gastroesophageal junction carcinomas: a critical insight. Crit Rev Oncol Hematol.

[16] Fontana E, Sclafani F, Cunningham D (2014). Anti-angiogenic therapies for advanced esophago-gastric cancer. Indian J Med Paediatr Oncol.

[17] Maeda K, Chung YS, Ogawa Y, Takatsuka S, Kang SM, Ogawa M (1996). Prognostic value of vascular endothelial growth factor expression in gastric carcinoma. Cancer.

[18] Juttner S, Wissmann C, Jons T, Vieth M, Hertel J, Gretschel S (2006). Vascular endothelial growth factor-D and its receptor VEGFR-3: two novel independent prognostic markers in gastric adenocarcinoma. J Clin Oncol.

[19] Suzuki S, Dobashi Y, Hatakeyama Y, Tajiri R, Fujimura T, Heldin CH (2010). Clinicopathological significance of platelet-derived growth factor (PDGF)-B and vascular endothelial growth factor-A expression, PDGF receptor-β phosphorylation, and microvessel density in gastric cancer. BMC Cancer.

[20] Stahlhut C, Suarez Y, Lu J, Mishima Y, Giraldez AJ (2012). miR-1 and miR-206 regulate angiogenesis by modulating VegfA expression in zebrafish. Development.

[21] Wang L, Chopp M, Gregg SR, Zhang RL, Teng H, Jiang A (2008). Neural progenitor cells treated with EPO induce angiogenesis through the production of VEGF. J Cereb Blood Flow Metab.

[22] Huang X, Stern DF, Zhao H (2016). Transcriptional profiles from paired normal samples offer complementary information on cancer patient survival-evidence from TCGA pan-cancer data. Sci Rep.

[23] Wu MH, Chen LM, Hsu HH, Lin JA, Lin YM, Tsai FJ (2013). Endothelin-1 enhances cell migration through COX-2 up-regulation in human chondrosarcoma. Biochim Biophys Acta.

[24] Wu MH, Lo JF, Kuo CH, Lin JA, Lin YM, Chen LM (2012). Endothelin-1 promotes MMP-13 production and migration in human chondrosarcoma cells through FAK/PI3K/Akt/mTOR pathways. J Cell Physiol.

[25] Chen X, Liang H, Zhang J, Zen K, Zhang CY (2012). Horizontal transfer of microRNAs: molecular mechanisms and clinical applications. Protein Cell.

[26] Bronisz A, Wang Y, Nowicki MO, Peruzzi P, Ansari KI, Ogawa D (2014). Extracellular vesicles modulate the glioblastoma microenvironment via a tumor suppression signaling network directed by miR-1. Cancer Res.

[27] Hudson RS, Yi M, Esposito D, Watkins SK, Hurwitz AA, Yfantis HG (2012). MicroRNA-1 is a candidate tumor suppressor and prognostic marker in human prostate cancer. Nucleic Acids Res.

[28] Migliore C, Martin V, Leoni VP, Restivo A, Atzori L, Petrelli A (2012). MiR-1 downregulation cooperates with MACC1 in promoting MET overexpression in human colon cancer. Clin Cancer Res.

[29] Minemura H, Takagi K, Miki Y, Shibahara Y, Nakagawa S, Ebata A (2015). Abnormal expression of miR-1 in breast carcinoma as a potent prognostic factor. Cancer Sci.

[30] Chen WS, Leung CM, Pan HW, Hu LY, Li SC, Ho MR (2012). Silencing of miR-1-1 and miR-133a-2 cluster expression by DNA hypermethylation in colorectal cancer. Oncol Rep.

[31] Pennacchietti S, Michieli P, Galluzzo M, Mazzone M, Giordano S, Comoglio PM (2003). Hypoxia promotes invasive growth by transcriptional activation of the met protooncogene. Cancer Cell.

[32] Puri N, Khramtsov A, Ahmed S, Nallasura V, Hetzel JT, Jagadeeswaran R (2007). A selective small molecule inhibitor of c-Met, PHA665752, inhibits tumorigenicity and angiogenesis in mouse lung cancer xenografts. Cancer Res.

[33] Han C, Zhou Y, An Q, Li F, Li D, Zhang X (2015). MicroRNA-1 (miR-1) inhibits gastric cancer cell proliferation and migration by targeting MET. Tumour Biol.

[34] Tsai KW, Hu LY, Chen TW, Li SC, Ho MR, Yu SY (2015). Emerging role of microRNAs in modulating endothelin-1 expression in gastric cancer. Oncol Rep.

[35] Lu J, Zhao FP, Peng Z, Zhang MW, Lin SX, Liang BJ (2014). EZH2 promotes angiogenesis through inhibition of miR-1/endothelin-1 axis in nasopharyngeal carcinoma. Oncotarget.

[36] Gherardi E, Birchmeier W, Birchmeier C, Vande Woude G (2012). Targeting MET in cancer: rationale and progress. Nat Rev Cancer.

[37] Wu MH, Huang PH, Hsieh M, Tsai CH, Chen HT, Tang CH (2016). Endothelin-1 promotes epithelial–mesenchymal transition in human chondrosarcoma cells by repressing miR-300. Oncotarget.

[38] Luo M, Hou L, Li J, Shao S, Huang S, Meng D (2016). VEGF/NRP-1axis promotes progression of breast cancer via enhancement of epithelial–mesenchymal transition and activation of NF-κB and β-catenin. Cancer Lett.

