# Supplementary material for: MicroRNA-1 acts as a tumor suppressor microRNA by inhibiting angiogenesis-related growth factors in human gastric cancer
Source: Gastric Cancer. 2017 May 10;21(1):41–54. doi: 10.1007/s10120-017-0721-x (PMC5741792; doi:10.1007/s10120-017-0721-x)
Supplement: Supplementary file 1 — Supplementary material 1 (PDF 149 kb) Online Resource 1. MiR-1 was the most markedly downregulated microRNA (miRNA) in gastric cancer (GC) according to the heatmap illustrating miRNome profiles from 295 gastric cancer cases from The Cancer Genome Atlas (TCGA). The color code represents log10 of the ratio of miRNA expression means of the tumor sample group and the normal tissue group. The brighter the green color, the greater the decrease in miRNA expression in the tumor sample group compared with the normal tissue group. The columns represent four different molecular subtypes of GC: Epstein–Barr virus infected (EBV), chromosomal instability (CIN), genomically stable (GS), and microsatellite instability (MSI). The rows denote different miRNAs [file 10120_2017_721_MOESM1_ESM.pdf]

Online Resource 1. miR-1 is the most markedly downregulated miRNA in gastric cancer according to the heatmap illustrating miRNomes profiles of 295 gastric cancer cases of TCGA

The Most Commonlly Differential Expressed miRNAs in GC (Data from TCGA)

Log10(Fold.Change) -1.013 … 1.964

	Class			
Mi R	EBV	CIN	GS	MSI
miR-1	-1.013	-0.999	-0.520	
miR-133a-3p	-0.931	-0.905	-0.394	
miR-451a	-0.555	-0.491	-0.579	
miR-145-5p	-0.633	-0.566	-0.328	
miR-9-5p	-0.639	-0.454	-0.409	
miR-139-5p	-0.598	-0.505	-0.320	
miR-486-5p	-0.455	-0.444	-0.473	
miR-145-3p	-0.508	-0.511	-0.203	
miR-29c-3p	-0.400	-0.434	-0.373	
miR-144-5p	-0.327	-0.311	-0.422	
miR-29b-2-5p	-0.315	-0.325	-0.317	
miR-365a-3p	-0.321	-0.278	-0.322	
miR-143-3p	-0.336	-0.401	-0.162	
miR-30a-3p	-0.385	-0.285	-0.196	
miR-27b-3p	-0.283	-0.177	-0.126	
miR-22-3p	0.095	0.058		0.138
miR-197-3p	0.186	0.103		0.202
miR-532-5p	0.589	0.441	0.179	
miR-20a-5p	0.515	0.334		0.448
miR-584-5p		0.507	0.280	0.532
miR-181a-1-5..	0.638	0.430	0.259	
miR-222-3p	0.451	0.408		0.471
miR-21-3p	0.555	0.405	0.338	0.548
miR-181a-1-3..		0.507	0.423	0.468
miR-141-5p	0.538	0.433		0.439
miR-185-5p	0.526	0.406		0.485
miR-146b-3p		0.388	0.457	0.581
miR-106b-3p	0.521	0.495		0.461
miR-93-5p	0.494	0.459		0.529
miR-1307-3p	0.489	0.449		0.579
miR-500a-3p	0.642	0.555	0.299	0.563
miR-17-5p	0.604	0.439		0.608
miR-708-3p	0.582		0.578	0.577
miR-194-5p		0.697	0.332	0.781
miR-182-5p	0.572	0.493		0.759
miR-146b-5p		0.528	0.557	0.756
miR-183-5p	0.643	0.570		0.788
miR-21-5p		0.671	0.642	0.732
miR-196b-5p		1.672	1.070	1.964
miR-196a-5p		1.846	1.304	1.739

Sum of Log10(Fold.Change) (color) broken down by Class vs. Mi R. The data is filtered on average of Log10(Fold.Change), which keeps all values. The view is filtered on Mi R, which keeps 127 of 127 members.
