# Supplementary figures and images for: MicroRNA-1 acts as a tumor suppressor microRNA by inhibiting angiogenesis-related growth factors in human gastric cancer
Source: Gastric Cancer. 2017 May 10;21(1):41–54. doi: 10.1007/s10120-017-0721-x (PMC5741792; doi:10.1007/s10120-017-0721-x)

**Online Resource 2.** miR-1 expression was negatively correlated with *VEGF-A*, *MET* and *EDN1*.

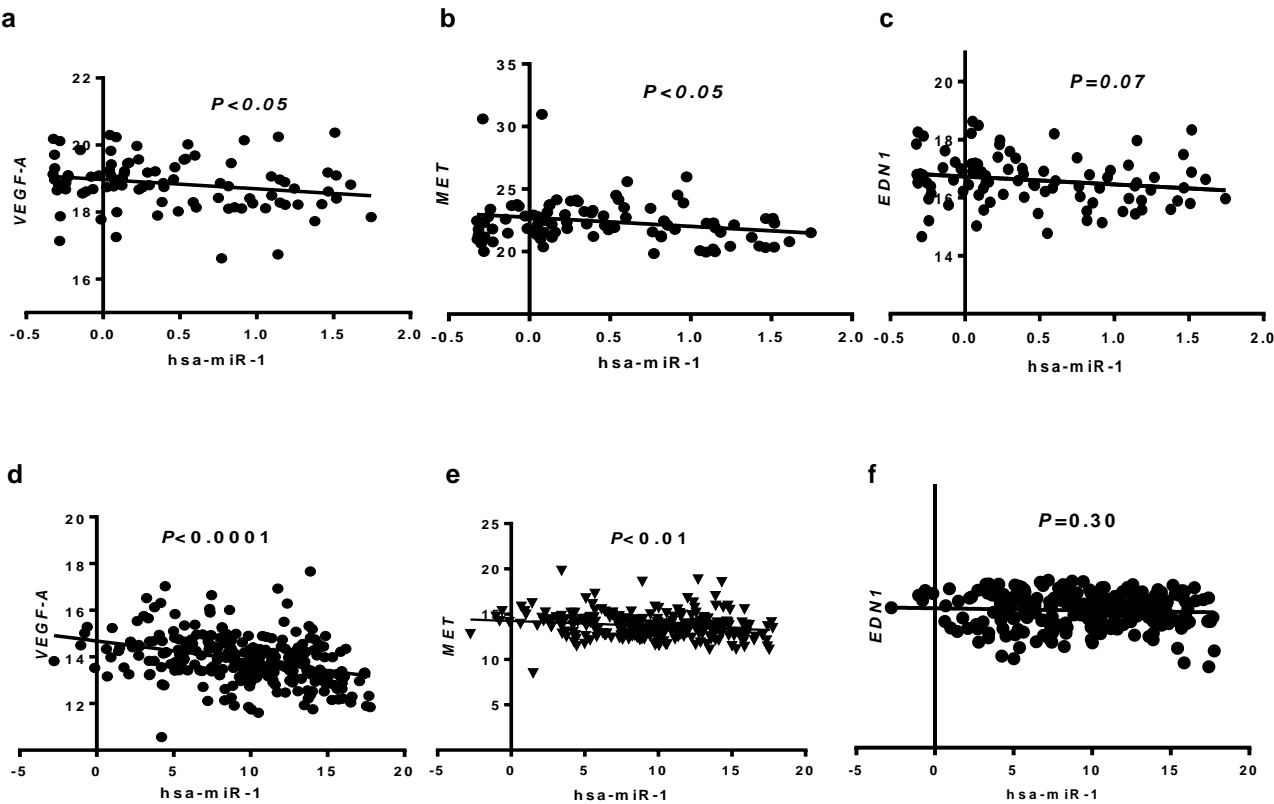

Supplement: Supplementary file 2 — Supplementary material 2 (PDF 188 kb) Online Resource 2. MiR-1 expression was negatively correlated with a VEGF-A [R = −0.23, 95% confidence interval (CI) −0.42 to −0.03], b MET (R = −0.21, 95% CI −0.40 to −0.01), and c EDN1 (R = −0.18, 95% CI −0.38 to 0.03) expression in Chinese patients with gastric cancer. MiR-1 expression also exhibited negative correlation with d VEGF-A (R = −0.35, 95% CI −0.46 to −0.24), e MET (R = −0.18, 95% CI −0.30 to −0.06), and f EDN1 (R = −0.06, 95% CI −0.18 to −0.0)) in the GC patients from The Cancer Genome Atlas cohort [file 10120_2017_721_MOESM2_ESM.pdf]
