# Supplementary material for: MicroRNA-1 acts as a tumor suppressor microRNA by inhibiting angiogenesis-related growth factors in human gastric cancer
Source: Gastric Cancer. 2017 May 10;21(1):41–54. doi: 10.1007/s10120-017-0721-x (PMC5741792; doi:10.1007/s10120-017-0721-x)

Online Resource 3. Inhibition of miR-1 increased angiogenesis related factors in both mRNA and protein levels

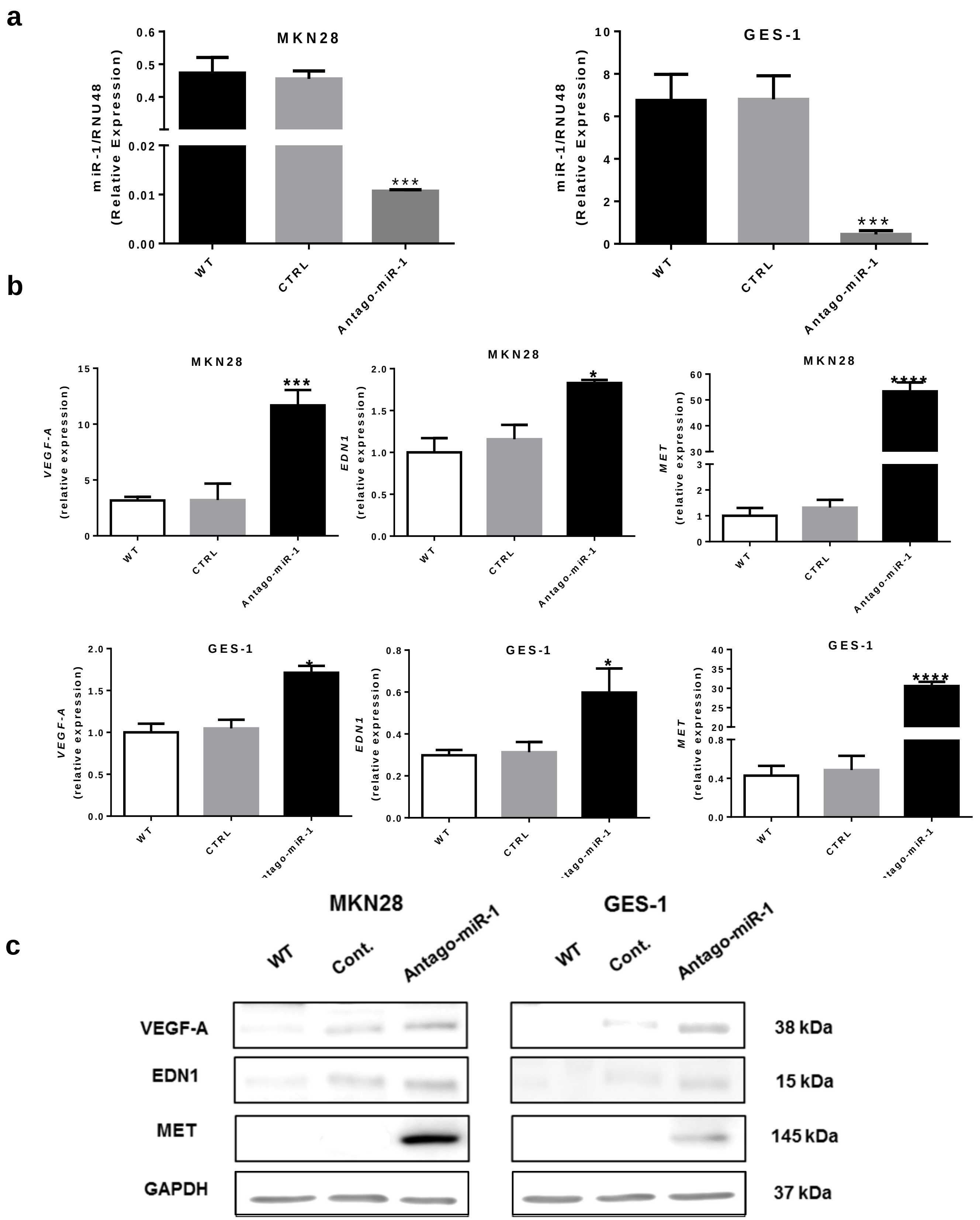

Supplement: Supplementary file 3 — Supplementary material 3 (PDF 69 kb) Online Resource 3. Inhibition of miR-1 increases expression of angiogenesis-related factors at both the messenger RNA level and the protein level. a Transfection with antago-miR-1 inhibited miR-1 expression in MKN28 and GES-1 cells. Mean ± standard deviation of three independent experiments. b Quantitative PCR assay. P values were determined by an unpaired two-sided t test. c Western blotting. Cont. control, CTRL, control, WT, wild type, *P < 0.05, **P < 0.01, ***P < 0.001, ****P < 0.0001 [file 10120_2017_721_MOESM3_ESM.pdf]
